# Effective Identification of Variety and Origin of Chenpi Using Hyperspectral Imaging Assisted with Chemometric Models

**DOI:** 10.3390/foods14111979

**Published:** 2025-06-03

**Authors:** Hangxiu Liu, Youyou Wang, Yiheng Wang, Jingyi Wang, Hanqing Hu, Xinyi Zhong, Qingjun Yuan, Jian Yang

**Affiliations:** 1 State Key Laboratory for Quality Ensurance and Sustainable Use of Dao-di Herbs, National Resource Center for Chinese Materia Medica, China Academy of Chinese Medical Sciences, Beijing 100700, China; hangxiuliu@163.com (H.L.); wangyouyou01@126.com (Y.W.); wyh@nrc.ac.cn (Y.W.); wangjingyi7108@163.com (J.W.); zhongxinyi0107@163.com (X.Z.); 2 Jiangxi Province Key Laboratory of Sustainable Utilization of Traditional Chinese Medicine Resources, Institute of Traditional Chinese Medicine Health Industry, China Academy of Chinese Medical Sciences, Nanchang 330115, China; 3 Dexing Research and Training Center of Chinese Medical Sciences, China Academy of Chinese Medical Sciences, Dexing 334213, China; 4 Jiangxi Health Industry Institute of Traditional Chinese Medicine, Nanchang 330115, China; 5 Research Center for Quality Evaluation of Dao-di Herbs, Ganjiang New District, Nanchang 330000, China; 6 Fruit Research Institute, Fujian Academy of Agricultural Sciences, Fuzhou 350013, China; hanqinghu@126.com

**Keywords:** Chenpi, variety and origin, rapid identification, hyperspectral imaging, machine learning

## Abstract

Geographical origins and varietal characteristics can significantly affect the quality of Citri Reticulatae Pericarpium (Chenpi), making rapid and accurate identification essential for consumer protection. To overcome the inefficiency and high cost of conventional detection methods, this study proposed a nondestructive approach that integrates hyperspectral imaging (HSI) with deep learning to classify Chenpi varieties and their geographical origins. Hyperspectral data were collected from 15 Chenpi varieties (citrus peel) across 13 major production regions in China using three dataset configurations: exocarp-facing-upward (Z), endocarp-facing-upward (F), and a fused dataset combining random orientations (ZF). Convolutional neural networks (CNNs) were developed and compared with conventional machine learning models, including partial least-squares discriminant analysis (PLS-DA), support vector machines (SVMs), and a multilayer perceptron (MLP). The CNN model achieved 96.39% accuracy for varietal classification with the ZF dataset, while the Z-PLSDA model optimized with second derivative (D2) preprocessing and competitive adaptive reweighted sampling (CARS) feature selection attained 91.67% accuracy in geographical origin discrimination. Feature wavelength selection strategies, such as CARS, simplified the model complexity while maintaining a classification performance comparable to that of the full-spectrum models. These findings demonstrated that HSI combined with deep learning could provide a rapid, nondestructive, and cost-effective solution for Chenpi quality assessment and origin traceability.

## 1. Introduction

Citrus fruits, including oranges, grapefruits, lemons, limes, tangerines, and mandarins, are among the most widely cultivated and consumed fruit crops globally [1], and they are valued for both their nutritional benefits and extensive industrial applications. Although a significant portion is consumed fresh, citrus processing generates substantial waste, primarily peels, seeds, and pulp, which serve as valuable raw materials for high-value industries such as essential oil extraction, citric acid production, and biofuel generation. Notably, residues account for nearly 50% of the processed fruit mass, with peels comprising the majority [2,3]. Citrus peels contain bioactive compounds, including polyphenols, flavonoids, essential oils, and dietary fibers, with significant applications in nutraceuticals, functional foods, and pharmaceuticals [4]. Therefore, efficient utilization of these discarded peels is crucial. In China, citrus fruits are highly valued in conventional Chinese medicine (TCM), contributing to well-known medicinal herbs, such as Chenpi, Foshou, Xiangyuan, and Zhiqiao [5,6]. Among these, Chenpi serves as the dried peel of mature *Citrus reticulata* Blanco (mandarin), which is listed in pharmacopoeias worldwide [7,8]. As the globally largest producer of mandarins, China produced 23.12 million tonnes in 2020, accounting for approximately 60% of the global production [9]. Transforming discarded citrus peels into Chenpi not only mitigates industrial waste but also enhances the supply of this highly valued dual-purpose TCM, which is widely used in both medicinal and culinary applications [10]. The rising market value of Chenpi continues to drive economic growth in this sector [11].

Despite the high value of Chenpi, the market faces several significant challenges, primarily due to the extensive diversity of mandarin varieties, which complicate unified and standardized processing. Several mandarin varieties, including *C. reticulata* ‘Chachi’, *C. reticulata* ‘Unshiu’, *C. reticulata* ‘Dahongpao’, and *C. reticulata* ‘Tangerina’, are officially listed in the Chinese Pharmacopoeia [7], while hundreds of other varieties, such as *C. reticulata* ‘Zhuhong’, *C. reticulata* ‘Ponkan’, and *C. reticulata* ‘Suavissima’, are also used for Chenpi production [12]. These varieties exhibit substantial differences in their active constituents and concentrations. For instance, *C. reticulata* ‘Dahongpao’ contains the significantly higher flavonoid levels compared to *Citrus grandis Tomentosa*, *Citrus ichangensis Swingle*, *Citrus sinensis* (L.) *Osbeck*, and *C. reticulata* ‘Buzhihuo’ [13], while *C. reticulata* ‘Chachi’ is particularly rich in terpenoids [14]. Such variations in bioactive compounds and flavor profiles pose considerable challenges for quality control in Chenpi, highlighting the need for rapid identification techniques to distinguish between these varieties and improve quality management and industrial efficiency. Furthermore, the composition and concentration of TCM are heavily influenced by geographical origin, with significant variations observed even within the same cultivar [15,16]. Major Chenpi production regions in China include Guangdong, Jiangxi, Sichuan, Fujian, and Zhejiang, with Guangdong accounting for 70% of the total Chenpi production, of which 90% originates from Xinhui [17]. Xinhui Chenpi derived from *C. reticulata* ‘Chachi’ grown in Xinhui County is widely regarded as the highest quality due to its protected geographical indication (PGI) status and the superior nutrient content [18,19]. In recent years, its commercial value has surged, which can be driven by the rising popularity of tea-related products such as “Xiao-Qing-Gan” and “Gan-Pu” tea, leading to the significant price disparities in the market [20] and contributing to an industry valued at 19 billion CNY (US$2.66 billion) in 2022 [21]. However, as the price of Xinhui Chenpi continues to rise, counterfeit products made from non-Xinhui mandarin varieties have flooded the herbal market, which can be falsely marketed as authentic Xinhui Chenpi. Consequently, accurately identifying both the origin and variety of Chenpi has become a critical challenge in ensuring product authenticity and maintaining market stability.

In the current Chenpi market, conventional detection methods rely primarily on sensory evaluation, physical analysis, and chemical testing [22]. However, the accuracy of sensory and physical assessments is often compromised [21] because of the striking similarities in mandarin peel appearance and the vast number of species, making precise identification of origin and variety highly challenging. Chemical analysis techniques, such as high-performance liquid chromatography (HPLC) [23], gas chromatography (GC) [9], and liquid chromatography–tandem mass spectrometry (LC-MS/MS) [24], allow precise differentiation by detecting the key constituents and volatile compounds in Chenpi [25]. However, these methods are inefficient, destructive, and time-consuming, and they require advanced technical expertise, rendering them impractical for onsite market screening. DNA barcoding technology has also been widely applied in herbal medicine authentication. However, conventional DNA barcoding techniques may be inadequate for Chenpi, which has an exceptionally long storage duration. DNA degradation over time can impair the polymerase chain reaction (PCR) efficiency, limiting its applicability [26]. Given these challenges, the development of rapid and accurate identification techniques to determine the origin and variety of Chenpi is essential to ensure quality control and authenticity verification.

Hyperspectral imaging (HSI) technology is widely used for food quality detection owing to its low cost, simple preprocessing, and ability to rapidly collect data from multiple samples [27]. When combined with conventional machine learning algorithms such as SVM and PLSR/PLSDA, HSI has been successfully applied to predict the chemical composition and origin of agricultural products [28,29]. However, conventional algorithms struggle to process large-scale data and typically rely on manual feature extraction [30,31]. In contrast, deep learning methods such as CNN and LSTM offer significant advantages in self-learning and efficiently handling large datasets [32,33], while integrating attention mechanisms (AMs) can further enhance prediction accuracy and efficiency [30,34]. Despite these advancements, no study has explored the combination of HSI and deep learning for predicting the origin and variety of Chenpi.

This study aimed to integrate deep learning with HSI technology, incorporating characteristic band selection and machine vision techniques to enhance the identification of the origin and variety of Chenpi. The primary research objectives were as follows: (1) to construct a comprehensive hyperspectral imaging database containing Chenpi samples from various origins and varieties; (2) to compare the predictive performance of different machine learning models and develop a hybrid deep learning model for predicting both geographic origin and variety; and (3) to develop and evaluate models specifically designed to predict the geographic origin and variety of Chenpi, with a particular focus on the same variety grown in different regions, especially those cultivated in PGI regions of China in order to assess the impact of geographic variability on prediction accuracy.

## 2. Materials and Methods

### 2.1. Sample Collection and Pretreatment

Mature mandarin fruits were harvested between September and December 2024 from eight provinces and 13 major production regions across China. Within each region, multiple sites were selected for subsample collection, resulting in 19 batches of mandarin fruit samples, with each batch comprising 60 individual fruits.

**Figure 1:**
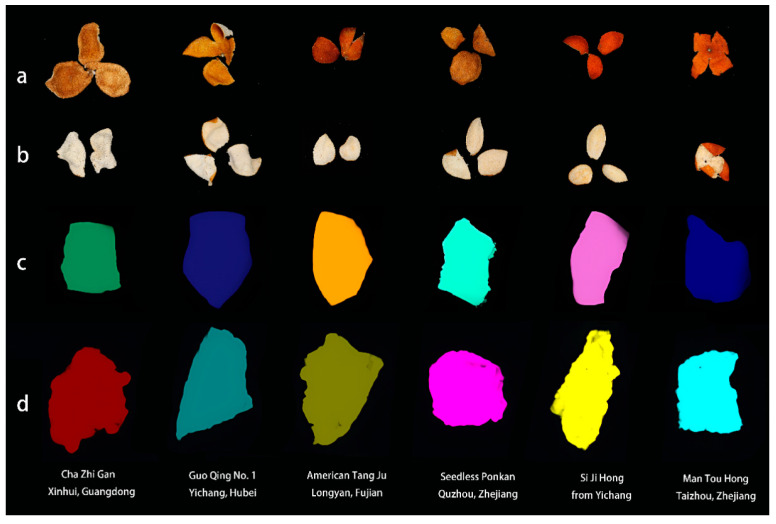
Partial samples of different varieties of Chenpi, divided into exocarp-facing-upward (Z) and endocarp-facing-upward (F). (**a**) Photograph of Z-side; (**b**) photograph of F-side; (**c**) ROI image of Z-side; (**d**) ROI image of F-side.

Following harvest, the fruit peels were naturally dried within the same year to produce Chenpi. The variety and geographical origin information of Chenpi are presented in Table 1. The sample set included 15 distinct Chenpi varieties collected from 13 geographical locations. The images of the exocarp and endocarp of some Chenpi variety samples are shown in Figure 1.

### 2.2. Hyperspectral Image Acquisition

Data were acquired using a HySpex-series hyperspectral imaging spectrometer (Norsk Elektro Optikk A/S, Oslo, Norway). The system comprised two imaging modules covering the visible-near-infrared (VNIR, SN0605; 410–990 nm) and short-wave infrared (SWIR, N3124; 950–2500 nm) spectral ranges, a halogen tungsten illumination source (H-LAM; Norsk Elektro Optikk, Oslo, Norway), a conveyor belt for sample transportation, and a computer for data acquisition. Operating with a spectral resolution of 6 nm, the system maintained a 25 cm working distance between the lens and samples, with the platform moving at a constant speed of 1.5 mm/s during the linear scanning. A small sample piece (approximately 15 × 25 mm) was extracted from each fruit for hyperspectral scanning on both the front and back surfaces, generating 2280 spectral datasets (19 batches × 60 fruit peels per batch × 2 scans per peel).

To reduce noise interference from dark currents and non-uniform illumination during HSI acquisition, the raw hyperspectral data underwent black-and-white reference calibration before further analysis [35]. This correction was applied using the following mathematical approach.

(1) $$R=\frac{R_{r}-R_{d}}{R_{w}-R_{d}}\times 100\%$$

where R denotes the corrected spectral data, R_r_ represents the raw spectral data, R_d_ denotes the dark reference data (obtained by turning off the illumination source and covering the camera lens to eliminate ambient light interference), and R_w_ represents the white reference data acquired from the spectrally flat calibration panel with the reflectance.

The spectral reflectance of individual Chenpi samples was extracted using thresholding and watershed segmentation methods. Specifically, the thresholding segmentation strategy is based on grayscale separation, where a threshold value of 0.2 is set to effectively distinguish and separate the Chenpi sample image from the background on the moving stage. According to the object size of Chenpi sample (with pixel size of VNIR for 1785–2973 and of SWIR for 31,076–39,745), the watershed algorithm is applied to achieve efficient segmentation between the whiteboard and the Chenpi sample. Also, the ROIs for representative samples of each orientation (F and Z) was added in the Figure 1c,d.

### 2.3. Spectral Preprocessing Methods

During hyperspectral imaging acquisition, the spectral data may contain extraneous information owing to noise, stray light, and baseline drift, which can negatively affect model performance. To enhance predictive accuracy and model stability, appropriate preprocessing of raw spectral data is typically required before modeling. In this study, five spectral preprocessing methods were applied and compared for their effects on classification performance: multiplicative scatter correction (MSC), first derivative (D1), second derivative (D2), Savitzky–Golay smoothing (SG), and standard normal variate (SNV).

### 2.4. Effective Wavelength Screening Algorithms

Full-spectrum hyperspectral data, with their wide spectral range, numerous bands, and large volume, can often lead to issues such as data redundancy and multicollinearity, increasing model complexity. Therefore, selecting characteristic spectral bands is essential to reduce data dimensionality, eliminate interference from irrelevant variables, and enhance both the efficiency and predictive performance of spectral modeling.

The Successive Projections Algorithm (SPA) uses projection operations in the vector space to eliminate irrelevant variables and address collinearity, identifying an optimal subset of wavelengths with minimal redundancy. SPA applies a sequential projection strategy to iteratively rank the initial wavelengths and generate multiple candidate subsets, from which the optimal subset is selected based on the model’s predictive performance [36]. In this study, the minimum and maximum numbers of selected bands were set to 10 and 50, respectively, using the preprocessing method configured as “autoscaling”.

The competitive adaptive reweighted sampling (CARS) method integrates Monte Carlo sampling with partial least-squares (PLS) regression coefficients to identify optimal feature variables. First, the candidate variables were screened by combining the PLS coefficients with an exponentially decreasing function, followed by adaptive reweighted sampling. The final wavelength combination was selected based on the lowest root mean squared error of cross-validation (RMSECV) through 10-fold cross-validation [37]. In this study, the number of Monte Carlo sampling iterations was set to 50, and the number of principal components was fixed at 10.

### 2.5. Conventional Machine Learning Model

Partial least-squares discriminant analysis (PLS-DA) is a high-dimensional linear classification model based on partial least-squares regression (PLSR) [38]. During modeling, the labeled numerical vectors were converted into dummy variable matrices, and predictions were made using the PLSR model by assigning samples to the class with the highest predicted value. To optimize the latent variables and prevent overfitting, a 10-fold cross-validation approach was applied with the number of latent variables limited to a maximum of 21.

A support vector machine (SVM) is a classifier suitable for handling non-linear data that maximizes the margin in the feature space and offers strong generalization capabilities. It determines an (N-1)-dimensional hyperplane as the decision boundary for classification in N-dimensional data, with the margin defined by the sum of distances between the hyperplane and support vectors on both sides, where larger margins correspond to a higher classification accuracy [39]. In this study, hyperparameters, including the kernel function type (e.g., polynomial kernel), polynomial degree (optimized range: 2–3), penalty coefficient *C* (10^−1^–10^2^), and kernel coefficient *g* (10^−7^–1), were optimized via a grid search. To ensure generalization, the optimal parameter combination was evaluated using leave-one-out cross-validation.

A multilayer perceptron (MLP) is a feedforward artificial neural network consisting of fully connected input, hidden, and output layers [40]. Trained by the backpropagation algorithm, it propagates errors backwards to update weights and biases, allowing the network to approximate target functions. Deep architectures with multiple hidden layers enhance the representational capacity. In this study, grid search optimization was used to determine the number of hidden layers, neurons per layer, and optimization algorithms (lbfgs, sgd, or Adam) with the activation function fixed as *ReLU*.

### 2.6. Deep Learning Model

As a leading deep learning (DL) architecture, convolutional neural networks (CNNs) have gained significant attention in both industry and academia, owing to their remarkable success in fields such as computer vision and natural language processing. A typical CNN consists of an input layer, convolutional layers, pooling layers, fully connected layers, and an output layer. This structure mimics biological neural networks with convolutional kernel scanning data to extract key features. In addition, CNNs employ a local weight-sharing mechanism that reduces parameter redundancy, thereby mitigating overfitting [41].

In this study, the CNN architecture was based on ResNet18 and consisted of eight convolutional layers, one pooling layer, and one fully connected layer. Convolutional operations were performed using predefined stride sizes and kernel dimensions to reduce data dimensionality. The ReLU activation function introduced nonlinearity, whereas the Adam optimizer (learning rate: 10^−4^) ensured rapid convergence during training. A Softmax classifier at the output layer was used to assign the spectral data to distinct categories. Additionally, L1/L2 regularization is applied to the models, introducing penalty terms in the loss function to control model complexity and prevent overfitting.

### 2.7. Data Analysis and Model Evaluation

The dataset was randomly divided into training, validation, and prediction sets at a 6:2:2 ratio (variety dataset: 900 samples, origin dataset: 300 samples), with the training set used for model fitting, the validation set for hyperparameter tuning, and the prediction set for performance evaluation. All spectral preprocessing and classification modeling were conducted using PyCharm (Python 3.10). PLS-DA, SVM, and MLP models were developed using the Scikit-learn library, whereas the CNN was implemented using the PyTorch deep learning framework (version 1.12.0+cu113).

Classification accuracy is the most commonly used metric for evaluating model performance, and it represents the ratio of correctly predicted samples to the total number of samples. Precision measures the ratio of correctly predicted positive samples among all predicted positive cases, while recall represents the ratio of correctly predicted positive cases to the actual total number of positive cases. Higher values approaching 100% indicate better classification performance. The confusion matrix provides a visual representation of the model performance, intuitively displaying key evaluation metrics such as accuracy, precision, and recall. The evaluation formula is as follows:

(2) $$Precision=\frac{TP}{TP+FP}\times 100\%,$$

(3) $$Recall=\frac{TP}{TP+FN}\times 100\%,$$

(4) $$Accuracy=\frac{TP+TN}{TP+TN+FP+FN}\times 100\%,$$

where *TP* denotes the number of true positive samples, *TN* is the number of true negative samples, *FP* is the number of false positive samples, and *FN* is the number of false negative samples.

## 3. Results

### 3.1. Raw Spectra of Chenpi Samples from Different Varieties and Origins

**Figure 2:**
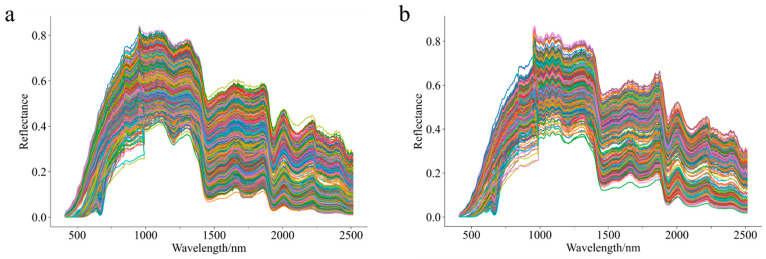
Spectral curves of Chenpi samples of different varieties and origins. (**a**) ZF dataset of variety. (**b**) ZF dataset of origin.

The spectral curves of Chenpi samples from different botanical varieties and geographical origins, from the ZF dataset that combines the Z and F datasets, are shown in Figure 2. While samples from different varieties and origins exhibit similar trends in spectral curves, differences in spectral reflectance intensity are observed, which may arise from variations in chemical composition between varieties and origins. The spectral curves display a sharp upward trend in the visible-near-infrared range (400–1000 nm). In the short-wave infrared range (1000–2500 nm), the curves show a fluctuating downward trend with distinct absorption peaks and troughs. A consistent trough at 500–700 nm reflected the color characteristics of the samples. The Z dataset spectral curves are more clustered and stable, whereas the F dataset exhibits greater noise and instability, requiring preprocessing methods to remove interference from excessive noise in the raw spectral curves.

A correlation analysis of spectral peaks revealed distinct molecular interactions: The absorption band near 1200 nm corresponds to the second stretching overtone of C–H bonds, predominantly originating from carbohydrate and lipid components. At 1300 nm, the spectral feature arises from C–H in-plane bending vibrations within organic matrices. The prominent peak at 1800 nm is attributed to O–H stretching vibrations associated with moisture absorption. Notably, although the peak positions of Chenpi from different varieties and origins are similar, there are significant differences in peak reflection intensity at the same location, indicating that spectral reflection patterns have different varieties and geographical characteristics.

### 3.2. Discriminant Analysis of Different Chenpi Varieties

In conventional machine learning approaches, five preprocessing methods (MSC, D1, D2, SG, and SNV) were applied to the raw spectra (RAW) to eliminate noise interference and baseline drift, followed by the development of PLS-DA, SVM, and MLP models based on the preprocessed data. In contrast, a convolutional neural network (CNN) leveraging its automated feature extraction capability can directly utilize raw spectra without preprocessing for effective feature learning. To assess the impact of sample orientation on classification performance, hyperspectral data were collected from Chenpi samples in two orientations: exocarp-facing-upward (Z) and endocarp-facing-upward (F). Additionally, a combined dataset (Z + F) was created by merging both orientations to simulate real-world scenarios, in which samples were randomly positioned during data acquisition.

The classification results for each model are listed in Table 2. Overall, except for SG preprocessing, most models showed improved classification accuracy after preprocessing, with D1 and SNV demonstrating universal applicability. Notably, the D1-MLP and SNV-SVM models consistently achieved accuracy rates above 90% across all three datasets (Z, F, and ZF), confirming their effectiveness in enhancing spectral feature discriminability. Among the four classification models, certain machine learning approaches (PLS-DA, SVM, and MLP) effectively distinguished Chenpi varieties, with most achieving over 80% accuracy in both the training and prediction sets. Specifically, the D1-MLP model attained the highest prediction accuracy (92.78%) for the Z dataset, whereas the SNV-SVM model achieved optimal performance (94.44% accuracy) on the F dataset. In contrast, the CNN demonstrated moderate classification performance on single-orientation datasets (Z or F) but outperformed all other models on the combined ZF dataset, achieving a prediction accuracy of 96.39% using the raw spectra. This result underscores the strong representational capacity of deep networks in handling multisource data fusion.

**Figure 3:**
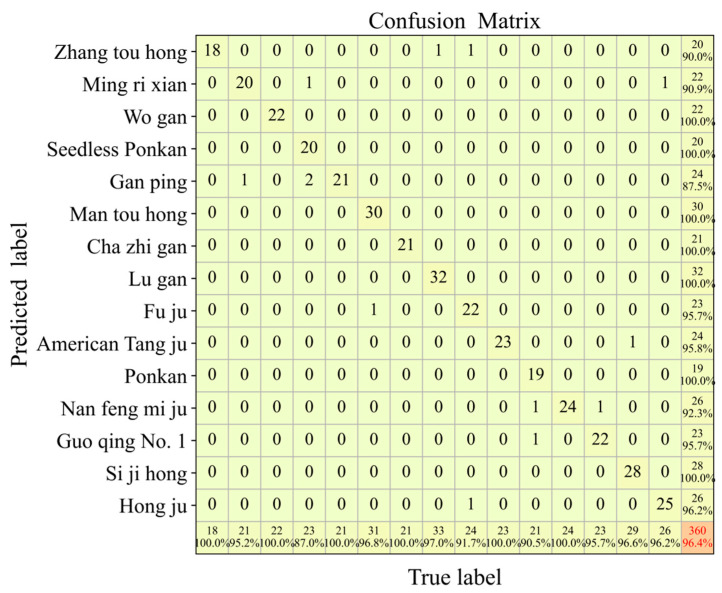
The confusion matrix on the prediction sets of the ZF-CNN.

To further assess the classification performance of the CNN model for each Chenpi variety, a confusion matrix (Figure 3) was constructed for the prediction set based on the optimal RAW-CNN model [42]. In the confusion matrix, the columns represent the true class labels and the rows indicate the predicted class labels. The diagonal cells show the number of correctly classified samples per class, with the rightmost column displaying the total number of true samples and their recall rates. The bottom row presents the number of predicted samples per class along with their precision rates. The results revealed that “Gan Ping” had a relatively high misclassification rate, achieving only 87.5% recall, while “Seedless Ponkan” exhibited reduced discriminability, with a precision of 87.0%. Most varieties demonstrated high precision and recall rates, particularly “Wo Gan” and “Cha Zhi Gan”, which achieved 100% recall and precision, indicating the strong distinctiveness in the model’s classification of these two varieties.

### 3.3. Discriminant Analysis of Different Chenpi Origins

In addition to varietal identification, tracing the geographical origin of Chenpi is a key challenge for market authentication. This study utilized the authentic Chenpi “Cha-Zhi-Gan” to develop origin discrimination models, following the same dataset partitioning protocol as previously described. The classification results are presented in Table 3.

Among the evaluated models, PLS-DA demonstrated robust performance in discriminating geographical origins, achieving classification accuracies above 80% across all datasets, with a prediction accuracy of 96.67% for the Z dataset. The SVM and MLP models exhibited significant variability depending on the preprocessing method, with only specific techniques yielding optimal results. The MLP model performed the best with D1 and D2 preprocessing, whereas the SVM model achieved the highest accuracy with SNV, reaching 95.00% and 94.17% for the F and ZF datasets, respectively. In contrast, the CNN model underperformed in origin identification, likely owing to the insufficient sample size, achieving an accuracy of approximately 80% across all datasets. Overall, the D2-PLSDA model applied to the Z dataset delivered the highest performance with training and prediction set accuracies of 98.75% and 96.67%, respectively.

**Figure 4:**
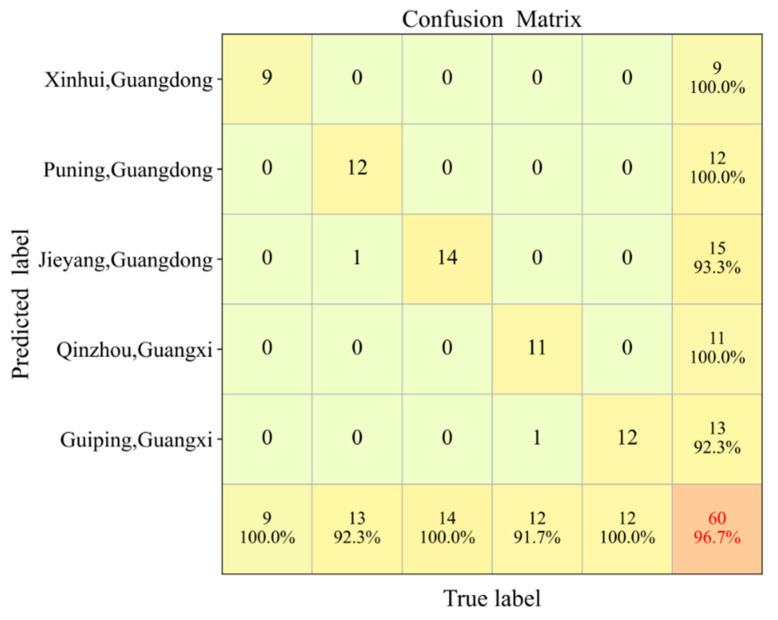
Confusion matrix on the prediction sets of the Z-D2-PLSDA.

To further evaluate the classification efficacy of the model for “Cha-Zhi-Gan” Chenpi in different geographical regions, a confusion matrix of the prediction set (Figure 4) was generated based on the optimal D2-PLSDA model. The results indicated that one sample from Guangdong Puning was misclassified as Guangdong Jieyang, and one from Guangxi Qinzhou was incorrectly predicted as Guangxi Guiping, indicating a higher likelihood of misclassification among geographically proximate origins. Notably, “Cha-Zhi-Gan” Chenpi from the authentic producing region of Guangdong Xinhui demonstrated absolute discriminative superiority, achieving 100% recall and precision, highlighting a clear spectral distinction between Xinhui origin Chenpi and those from other regions.

### 3.4. Extraction of Spectral Feature Wavelength

**Figure 5:**
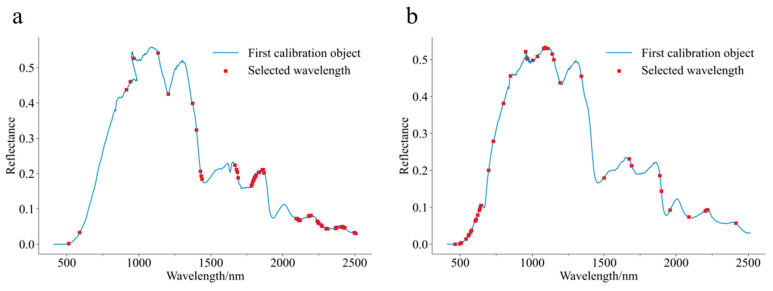
Selected wavelengths for best classification model of Chenpi by CARS. (**a**) ZF-CARS-CNN; (**b**) Z-D2-CARS-PLSDA.

To determine the most effective wavelength screening method for model simplification, SPA and CARS were used to extract the feature wavelengths from the ZF-CNN model (optimal for varietal classification) and the Z-D2-PLSDA model (optimal for origin discrimination) based on their full-spectrum classification results. The outcomes are summarized in Table 4 and Figure 5.

The number of feature wavelengths selected via the SPA (20–38) was significantly lower than that selected via the CARS (41–57). Both methods resulted in reduced model accuracy compared with full-spectrum modeling, likely owing to the loss of informative wavelengths during dimensionality reduction. The ZF-CNN model was particularly sensitive to wavelength reduction, with prediction accuracies dropping to 73.06% (SPA) and 85.00% (CARS). In contrast, the Z-D2-PLSDA model combined with CARS demonstrated strong performance, retaining a prediction accuracy of 91.67% using only 41 feature wavelengths, achieving an accuracy comparable to that of the full-spectrum model, while significantly reducing the complexity. CARS outperformed SPA in feature wavelength selection by preserving critical spectral information despite retaining more wavelengths and effectively balancing accuracy and error minimization. These findings suggest that the adaptive reweighting mechanism of CARS mitigates information loss more effectively than the projection-based approach of SPA, particularly in scenarios that require high discriminative fidelity.

## 4. Discussion

This study explored the classification of Chenpi from distinct varieties and geographical origins (“Cha-Zhi-Gan”) using HSI and machine learning models. Three sample orientation configurations were evaluated: exocarp-facing-upward (Z), endocarp-facing-upward (F), and a fused dataset combining random orientations (ZF). The classification models constructed from these configurations exhibited varying performance. Among the machine learning approaches (PLS-DA, SVM, and MLP), the Z dataset achieved a slightly higher classification accuracy than the F and ZF configurations. Contrary to the expected benefits of data fusion, the ZF approach underperformed, likely owing to redundant or noisy spectral information from the F orientation, which diminished the model’s discriminative capacity.

This study compared the deep learning model CNN with conventional machine learning models (PLS-DA, SVM, and MLP), highlighting the superior performance of CNN under large-sample conditions [43]. In the varietal classification of Chenpi peels using CNN, the fused ZF dataset group significantly improved the accuracy to 96.39%, establishing CNN as the optimal model for varietal discrimination. In the geographical origin classification, the CNN attained prediction accuracies of 80.00% (Z dataset) and 75.00% (F dataset), with a modest increase to 85.83% for the ZF dataset. This limited improvement may be attributed to the smaller sample size for origin discrimination (300 samples per single-orientation dataset, 600 total after fusion), further confirming the CNN’s sensitivity to sample quantity and its impact on model performance [44]. Overall, CNN and machine learning models exhibit complementary strengths. Compared with conventional approaches, CNN’s multilayered neural network architecture enables robust feature extraction and high representational capacity, making it particularly effective in large-sample scenarios. Although CNN requires a longer computational time for training, its advantages in terms of generalizability, stability, and interpretability surpass those of conventional models [45], making it highly effective for Chenpi varietal discrimination.

A comparative analysis of the two feature wavelength selection methods highlighted the superiority of CARS over SPA. Although SPA selected fewer characteristic wavelengths, the resulting model exhibited significant performance deterioration, suggesting that the selected wavelengths contained limited informative content. In contrast, CARS delivered more robust results, with its optimal model, Z-D2-PLSDA, constructed using 57 characteristic wavelengths, achieving a prediction set accuracy of 91.67%. By leveraging the PLS regression coefficients, CARS iteratively selected the wavelengths contributing the most to the model while dynamically adjusting the weights. Despite its higher computational complexity and a larger number of selected wavelengths, CARS more effectively identified the critical wavelengths, ultimately enhancing the model performance.

The feature wavelength selection curves indicated that ZF-CARS-CNN predominantly selected the characteristic bands in the near-infrared range, suggesting that front and back surface data modeling prioritized the near-infrared bands with richer information. In contrast, Z-D2-CARS-PLSDA primarily selected wavelengths within the 500–700 nm visible light range, which contained abundant color information, highlighting that focusing on visible light wavelengths during Z dataset modeling could yield superior results.

## 5. Conclusions

Geographical differences and varietal characteristics significantly influence the quality indicators of Chenpi, making accurate identification essential for protecting consumer rights. Moreover, there is an urgent need in food research for efficient and nondestructive rapid detection technologies to assess food quality. As a promising nondestructive detection technique, HSI has been widely applied in food and agricultural product quality evaluation. This study integrated HSI with deep learning to assess Chenpi samples from different varieties and geographical origins to develop a classification model for rapid differentiation. By incorporating the feature wavelength selection strategies, the model was significantly simplified while maintaining a classification performance comparable to that of full-wavelength models.

The results demonstrated that integrating HSI technology with deep learning provided a rapid and nondestructive approach to predicting Chenpi varieties and origins. Future research could expand the range of Chenpi varieties and origins while incorporating incremental learning to enhance model applicability and generalization. This approach could facilitate large-scale adoption of hyperspectral imaging for origin tracing and quality evaluation of conventional Chinese medicinal materials, particularly Chenpi.

## Figures and Tables

**Table 1:** Geographical scale information of different varieties of Chenpi.

No.	Chenpi Variety	Geographical Origin
C-1	Zhang tou hong	Xingan, Jiangxi
C-2	Ming ri xian	Quzhou, Zhejiang
C-3	Wo gan	Quzhou, Zhejiang
C-4	Seedless Ponkan	Quzhou, Zhejiang
C-5	Gan ping	Quzhou, Zhejiang
C-6	Man tou hong	Taizhou, Zhejiang
C-7, C-8, C-9	Cha zhi gan	Xinhui, Puning and Jieyang, Guangdong
C-10, C-11	Cha zhi gan	Qinzhou and Guiping, Guangxi
C-12	Lu gan	Fuzhou, Fujian
C-13	Fu ju	Fuzhou, Fujian
C-14	American Tang ju	Longyan, Fujian
C-15	Ponkan	Tujia and Miao Autonomous Prefecture, Hunan
C-16	Nan feng mi ju	Yichang, Hubei
C-17	Guo qing No. 1	Yichang, Hubei
C-18	Si ji hong	Yichang, Hubei
C-19	Hong ju	Wanzhou, Chongqing

**Table 2:** The accuracy of discriminant analysis models for different Chenpi varieties.

Models	Pretreatments	Z			F			ZF		
		Training Set (%)	Validation Set (%)	Prediction Set (%)	Training Set (%)	Validation Set (%)	Prediction Set (%)	Training Set (%)	Validation Set (%)	Prediction Set (%)
PLS-DA	RAW	86.81	85.38	79.44	90.14	89.12	90.56	86.25	86.19	85.28
	MSC	90.56	86.79	85.56	91.11	91.01	91.67	87.64	84.44	84.17
	D1	93.33	88.23	87.78	92.36	89.24	90.56	92.57	90.28	90.28
	D2	96.67	91.24	90.00	87.78	84.67	83.89	89.38	83.33	83.89
	SG	87.22	84.17	80.56	91.25	90.14	91.67	86.04	84.34	85.28
	SNV	90.42	86.42	85.56	90.14	90.00	87.78	88.12	85.56	85.28
SVM	RAW	93.19	87.33	84.44	86.39	83.06	76.67	83.75	81.24	80.00
	MSC	91.67	84.37	85.00	84.44	82.24	81.11	83.13	82.56	78.89
	D1	96.81	90.28	88.33	98.19	93.36	92.22	97.57	93.79	94.44
	D2	97.64	88.67	87.22	99.44	86.34	85.56	99.03	93.04	92.22
	SG	90.83	85.00	83.33	81.81	74.56	73.33	79.58	78.56	74.44
	SNV	99.58	95.84	90.56	99.86	95.66	94.44	98.82	91.39	92.78
MLP	RAW	90.00	85.76	82.78	70.96	70.12	70.56	76.60	75.67	71.94
	MSC	86.11	80.12	80.56	65.28	64.36	62.78	73.82	72.22	70.28
	D1	100.0	93.24	92.78	99.72	94.12	93.33	98.68	94.12	94.72
	D2	97.92	93.45	92.22	81.67	80.33	77.22	84.72	83.33	81.39
	SG	81.81	75.67	73.33	65.14	63.44	62.22	61.46	60.39	54.44
	SNV	94.31	87.42	85.00	94.44	89.33	86.11	84.31	82.12	83.06
CNN	RAW	99.70	85.56	83.33	98.08	90.23	88.33	99.18	98.67	96.39

**Table 3:** The accuracy of discriminant analysis models for different “Cha zhi gan” Chenpi origins.

Models	Pretreatments	Z			F			ZF		
		Training Set (%)	Validation Set (%)	Prediction Set (%)	Training Set (%)	Validation Set (%)	Prediction Set (%)	Training Set (%)	Validation Set (%)	Prediction Set (%)
PLS-DA	RAW	90.83	85.42	86.67	96.67	92.42	83.33	85.83	81.42	83.33
	MSC	96.25	95.00	88.33	93.75	91.67	93.33	87.29	85.83	82.50
	D1	98.97	98.33	93.33	95.00	93.67	91.67	96.25	91.67	90.83
	D2	98.75	95.75	96.67	88.33	82.50	85.00	96.04	92.42	90.83
	SG	88.33	87.29	83.33	90.42	89.33	85.00	85.42	82.50	85.00
	SNV	95.83	92.83	90.00	97.08	87.42	85.00	85.42	81.63	83.33
SVM	RAW	84.17	83.67	83.33	85.83	83.33	76.67	81.04	79.58	79.17
	MSC	85.00	80.00	78.33	82.92	81.67	78.33	81.87	75.00	77.50
	D1	92.08	86.67	85.00	96.67	89.02	85.00	93.54	90.21	86.67
	D2	92.08	88.33	91.67	95.00	84.58	87.29	93.13	84.79	85.00
	SG	82.50	80.33	78.33	82.92	76.67	75.00	76.46	74.38	75.00
	SNV	98.75	93.75	91.67	99.17	95.42	95.00	97.92	95.63	94.17
MLP	RAW	78.33	75.00	76.67	73.75	72.33	70.00	92.08	90.33	85.83
	MSC	78.75	73.33	70.00	90.83	90.00	80.00	95.83	90.79	87.50
	D1	95.00	92.75	95.00	93.33	85.83	80.00	99.38	95.75	91.67
	D2	98.75	91.67	93.33	88.33	87.67	83.33	98.75	90.17	91.67
	SG	80.83	72.67	70.00	73.33	72.42	71.67	98.75	86.67	84.17
	SNV	81.67	78.75	76.67	92.08	85.33	78.33	95.83	82.50	83.33
CNN	RAW	83.33	78.33	80.00	77.50	76.67	75.00	89.00	87.90	85.83

**Table 4:** The accuracy of optimal classification model for Chenpi varieties and origins based on feature wavelength selection using different methods.

**Models**	**Methods**	**Number**	**Training** **Set (%)**	**Validation** **Set (%)**	**Prediction** **Set (%)**
ZF-CNN	SPA	38	77.19	74.44	73.06
	CARS	57	88.67	86.78	85.00
Z-D2-PLSDA	SPA	20	76.67	72.34	71.67
	CARS	41	92.92	92.67	91.67

## Data Availability

The original contributions presented in the study are included in the article, further inquiries can be directed to the corresponding author.
